# Supplementary material for: The causal role of circulating inflammatory markers in osteoporosis: a bidirectional Mendelian randomized study
Source: Front Immunol. 2024 Jul 18;15:1412298. doi: 10.3389/fimmu.2024.1412298 (PMC11291241; doi:10.3389/fimmu.2024.1412298)
Supplement: Supplementary file 1 [file DataSheet_1.zip › supplementary material/OSTEOPOROSIS-CIM/GCST90274758.tsv.gz_leave_one_out.pdf]

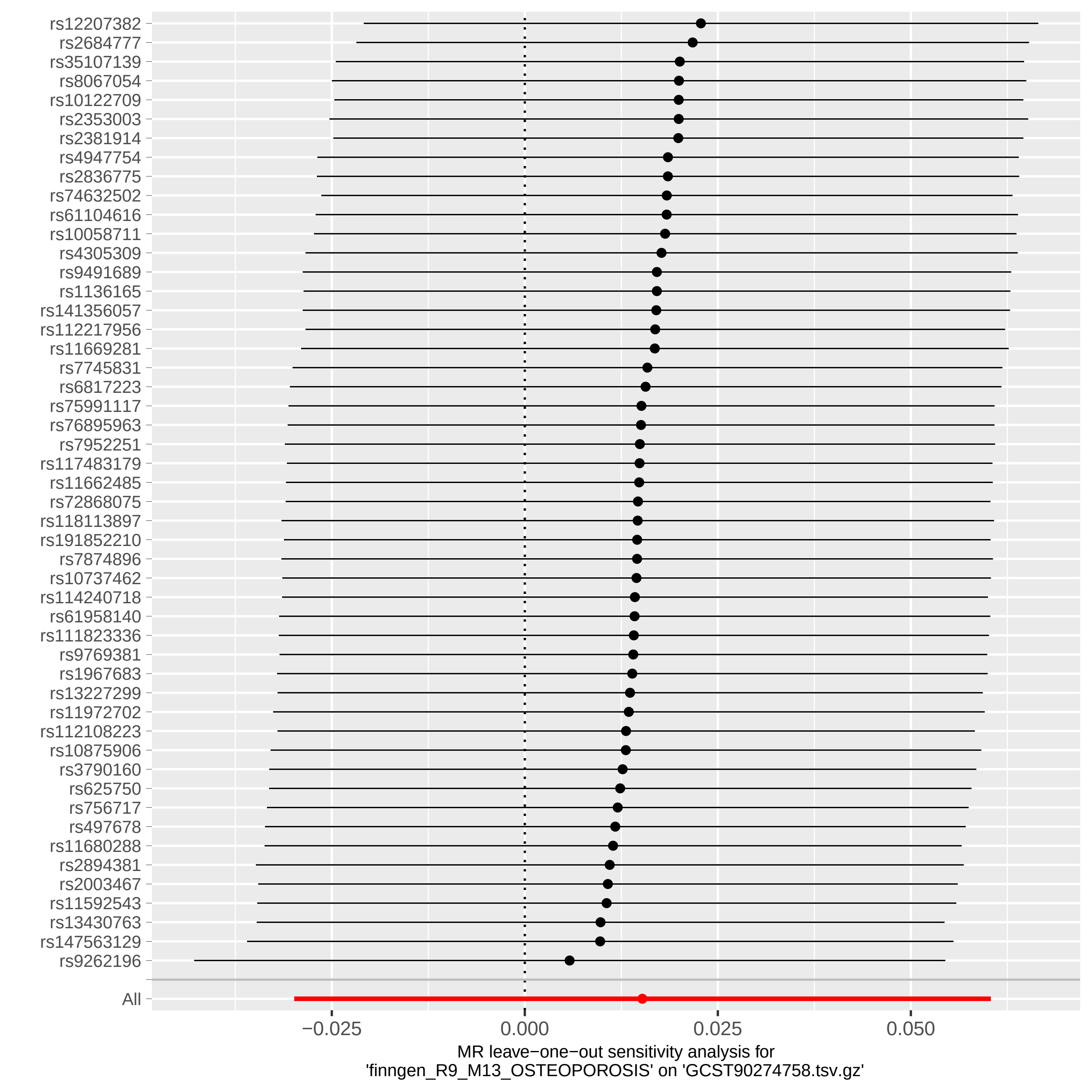
rs12207382
rs2684777
rs35107139
rs8067054
rs10122709
rs2353003
rs2381914
rs4947754
rs2836775
rs74632502
rs61104616
rs10058711
rs4305309
rs9491689
rs1136165
rs141356057
rs112217956
rs11669281
rs7745831
rs6817223
rs75991117
rs76895963
rs7952251
rs117483179
rs11662485
rs72868075
rs118113897
rs191852210
rs7874896
rs10737462
rs114240718
rs61958140
rs111823336
rs9769381
rs1967683
rs13227299
rs11972702
rs112108223
rs10875906
rs3790160
rs625750
rs756717
rs497678
rs11680288
rs2894381
rs2003467
rs11592543
rs13430763
rs147563129
rs9262196
All
−0.025
0.000
0.025
0.050
MR leave−one−out sensitivity analysis for
'finngen_R9_M13_OSTEOPOROSIS' on 'GCST90274758.tsv.gz'
